# Study Protocol for a Prospective Longitudinal Cohort Study to Identify Proteomic Predictors of Pluripotent Risk for Mental Illness: The Seoul Pluripotent Risk for Mental Illness Study

**DOI:** 10.3389/fpsyt.2020.00340

**Published:** 2020-04-21

**Authors:** Tae Young Lee, Junhee Lee, Hyun Ju Lee, Yunna Lee, Sang Jin Rhee, Dong Yeon Park, Myung Jae Paek, Eun Young Kim, Euitae Kim, Sungwon Roh, Hee Yeon Jung, Minah Kim, Se Hyun Kim, Dohyun Han, Yong Min Ahn, Kyooseob Ha, Jun Soo Kwon

**Affiliations:** ^1^Department of Neuropsychiatry, Pusan National University Yangsan Hospital, Yangsan, South Korea; ^2^Department of Neuropsychiatry, Seoul National University Hospital, Seoul, South Korea; ^3^Department of Neuropsychiatry, Kosin University Gospel Hospital, Pusan, South Korea; ^4^Department of Psychiatry, National Center for Mental Health, Seoul, South Korea; ^5^Department of Psychiatry, The Armed Forces Capital Hospital, Seongnam, South Korea; ^6^Department of Neuropsychiatry, Seoul National University Bundang Hospital, Seongnam, South Korea; ^7^Department of Neuropsychiatry, Hanyang University Hospital, Seoul, South Korea; ^8^Department of Psychiatry, SMG-SNU Boramae Medical Center, Seoul, South Korea

**Keywords:** bipolar disorder, high-risk for mental illness, major depressive disorder, pluripotential, proteomics, schizophrenia, transdiagnostic

## Abstract

**Background:**

The Seoul Pluripotent Risk for Mental Illness (SPRIM) study was designed to identify predictors leading to mental illness in help-seeking individuals by securing sufficient statistical power through transdiagnostic approaches. The SPRIM study aims to examine the clinical characteristics of high-risk individuals for mental illness and to identify proteomic biomarkers that can predict the onset of mental illness.

**Methods:**

This paper describes the study protocol of the SPRIM study. We aim to recruit 150 participants who meet the criteria for high risk for major mental illness, 150 patients with major psychiatric disorders (schizophrenia, bipolar disorder, and major depressive disorder), and 50 matched healthy control subjects for 2 years. Clinical evaluations, self-report measures, and proteomic analyses will be implemented. The assessment points are at baseline, 6, 12, 18, and 24 months.

**Conclusions:**

In the present study, we introduced the study protocol of the SPRIM study, which is the first prospective cohort study of transdiagnostic high-risk concepts using proteomic biomarkers. This study has a paradigm that encompasses various diseases without aiming at predicting and preventing the development of a specific mental illness in help-seeking individuals. The transdiagnostic high-risk concept could be extended to provide a perspective for people with various psychopathological tendencies below a threshold, such that they do not meet the existing diagnostic criteria of mental illnesses, to determine what may lead them to a specific disease and help identify appropriate preventative interventions.

## Introduction

For the past 20 years, the concept of ultra/clinical high-risk for psychosis (CHR-P) has been proposed and developed for the early detection and prevention of the transition to psychosis (1–4). Previous studies have shown good predictability compared with other medical diseases, and it was expected that effective interventions for early psychosis would soon be available (5). However, as time went on, the incidence of psychotic transition in CHR-P was found to gradually decrease in several cohort studies, and the reasons, such as lead-time bias due to early referral, high rate of false positives, and prevention through effective treatments, have been discussed (6–8). Recent studies have revealed that CHR-P is not pluripotent but highly specific for psychosis, and the problem of comorbidity is not significantly affected (9, 10), however since the necessity of specialized clinics with trained experts, we realized that the current high-risk concept covers only a fraction of the total schizophrenia incidence (11, 12). This epidemiologic inadequacy is also supported by the fact that psychosis still develops, although at a low rate, in clinical high-risk for non-psychotic mental disorders (13). Lack of the pragmatic transdiagnostic ability of the CHR-P designation and modest statistical power due to the recruitment difficulties and low conversion rate do not given much latitude in high-risk research (14, 15). As a way to overcome the limitation of statistical power, a method of expanding to a broad range of disorders has been proposed instead of limiting the outcome to schizophrenia. Recently, McGorry and his colleagues presented the CHARMS approach, a pioneering new concept, which is a transdiagnostic cohort program (16). This program is a high-risk program that covers a variety of outcomes, including psychotic disorder, bipolar disorder, depressive disorder, etc., unlike the existing CHR cohort program aimed at the onset of psychosis. The CHARMS approach is somewhat different from the current CHR concept that targets only schizophrenia and more closely reflects the clinical staging model that distinguishes a distress that needs only a little help or a stage that requires clinical attention just before transitioning over the threshold on the continuum of mental illness (17, 18). Despite the fact that there are already other high-risk approaches such as the bipolar prodrome, it is expected that the high-risk studies adopting this clinical staging perspective will provide a transdiagnostic understanding of mental illnesses (19–21). Although there have been many studies on the transition and remission in high-risk populations, few studies have been conducted using the definition of transdiagnostic high risk and investigated the long-term outcomes from this approach (22–24). However, this approach is a more common practice in the community because it is more practical for addressing mental illness that can be performed under normal circumstances and does not need a specialized clinic. The cohort program for a broad range of mental disorders is expected to provide greater flexibility in finding biomarkers to predict the development of mental disorders based on higher statistical power by recruiting a wider range of subjects.

The identification of biomarkers in mental illness plays an essential role in the differential diagnosis of disease and prediction of prognosis (25, 26). In psychiatry, which is based on phenotypic classification, biomarker research has a very long history (27, 28). In high-risk for psychosis, meta-analytical sequential testing simulations showed that probabilistic risk assessment using the information from clinical assessment has a greater predictive accuracy compared with that of the phenotypical model only (29). Moreover, considering phenotypical nondisjunction and biotypical reclassification of schizophrenia spectrum disorders or schizophrenia and bipolar disorder, applying the current high-risk concepts based on the existing classification systems of mental illness makes it more difficult to predict outcomes (30, 31). Therefore, a transdiagnostic approach with biomarkers would be a prerequisite for more accurate diagnosis and prognosis prediction. Recently, biomarker studies using proteomics have gained more attention (32, 33). Proteomics generally refers to the large-scale experimental analysis of proteins and proteomes; it has the advantage of being able to identify alterations in certain steps of the biochemical pathways of mental illness because it can distinguish the overexpression of specific proteins (34). Proteomic analyses are used to identify disease-specific alterations, including those for schizophrenia, bipolar disorder, and major depressive disorder (35). Furthermore, proteomics profiling has revealed that inflammatory biomarkers may serve as predictors of antidepressant treatment response (36). Besides, data-driven proteomic analysis using feature selection is expected to provide a new basis for the transdiagnostic approach (37, 38).

Since proteomic analysis is performed through blood sampling, it can be performed more easily than other biomarker studies, including neuroimaging or electrophysiology. However, no studies have used proteomics analyses in a high-risk group before the onse of the disease. Therefore, studies using proteomic biomarkers in a high-risk population before a transition to mental illnesses will enable further classification and prediction at the molecular level.

In this prospective cohort study, we have three goals. First, we will examine the clinical characteristics and clinical outcomes for subjects who do not meet the diagnostic criteria for mental illness, and these subjects will be classified as high-risk individuals for specific psychiatric disorders (schizophrenia, bipolar disorder, major depressive disorder, and undifferentiated). Second, we will identify proteomic biomarkers that differentiate (1) patients with mental illnesses and healthy individuals, and (2) patients with schizophrenia, bipolar disorder, and major depressive disorder. Third, we will attempt to explore the proteomic markers that predict the transition of the high-risk group to mental illness.

## Methods

### Design

The Seoul Pluripotent Risk for Mental Illness (SPRIM) study is a prospective cohort study and aims to recruit 150 participants who meet the criteria for high risk for major mental illness for 2 years. To verify disease-specific proteomic profiles and to use them as templates for classifying and predicting the course of high-risk individuals, we will also recruit 150 patients with major psychiatric disorders (schizophrenia, bipolar disorder, major depressive disorder) and 50 matched, healthy control subjects.

Participants are being recruited from the Seoul National University Hospital, Seoul National University Bundang Hospital, SMG-SNU Boramae Medical Center, Hanyang University Hospital, Inha University Hospital, National Mental Health Center, Korean Armed Forces Capital Hospital, and Gwanak-gu Public Health Center. All institutions are located in Seoul, covering community, military service, and general hospital populations. All participants are referred to the Seoul Youth Clinic or Mood Disorders Clinic of Seoul National University Hospital and evaluated by experienced psychiatrists.

### Sample

Potential participants are high-risk individuals for major mental disorders aged 15–45 who were recruited and referred to Seoul National University Hospital. High-risk subjects participating in the SPRIM study are classified into four categories. The inclusion criteria for each group are shown in **Table 1**. Patients with major mental disorders (schizophrenia, bipolar disorder, and major depressive disorder) who had been diagnosed within the previous 5 years are also being recruited from the Seoul National University Hospital. The exclusion criteria are as follows: the presence of mental retardation, a pervasive developmental disorder, neurological disorders, a history of head trauma with loss of consciousness, pregnancy, medical conditions that may cause mental illness, and substance abuse.

**Table 1:** SPRIM study criteria.

Subgroup	Description
**Psychosis risk group**	15–45 years old Attenuated psychosis group. Meeting all of the following criteria: (1) At least one SOPS score rated 3–5 points from PSP1 to PSP5; (2) Symptoms occurred during the past years, and the current score is higher than 12 months ago; (3) Symptoms appear at least once a week in the past month. Brief limited intermittent psychotic symptoms group. Meeting all of the following criteria: (1) At least one of SOPS score of 6 points was obtained from PSP1 to PSP5; (2) Symptoms have started within the last 3 months; (3) Symptoms now appear more than once a month, at least several minutes per day. Exclusion criteria: antipsychotics are prescribed for more than 30 days.
**Bipolar risk group**	15–45 years old Common inclusion criteria: Meeting criteria for at least one group from the below in the last 12 months. Subthreshold mania group: manic episodes lasting more than two days, less than four days. Depression + cyclothymic features group: Meeting all of the following criteria: (1) at least a week, there is depressed mood or loss of interest or pleasure; (2) two or more items from diagnostic criteria A of major depressive disorder in DSM; (3) multiple episodes with subthreshold manic symptoms not meeting group I criteria and numerous episodes with depressive symptoms. Depression + genetic risk group: The criteria for depression listed above AND the presence of first-degree relatives with bipolar disorder. Cyclothymic features and genetic risk group: Meeting all of the following criteria: (1) Numerous episodes with subthreshold manic symptoms, not meeting group I criteria and numerous episodes with depressive symptoms; (2) the presence of first-degree relatives with bipolar disorder. Subthreshold mixed episode group: Meeting all of the following criteria: (1) Subthreshold mania; (2) depressed mood nearly every day but less than five consecutive days. Mood swings group: There is a recent onset of mood instability. Exclusion criteria: (1) history of psychosis that lasted more than seven days in SIPS evaluation regardless of treatment; (2) history of mood stabilizer treatment for six weeks or more; (3) history of antipsychotic treatment for more than three weeks, and (4) history of bipolar I disorder. Extended bipolar risk group: Participants who have been diagnosed with the following mental illness before the age of 25 and has not yet passed 5 years; bipolar II disorder, cyclothymia, bipolar disorder NOS, recurrent MDD (recurrent MDD, regardless of the duration after diagnosis in case of recurrent MDD).
**Depression risk group**	15–45 years old Meeting all of the following criteria in the last 12 months: (1) at least 1 week of a depressed mood or loss of interest or pleasure; (2) two or more items on diagnostic criteria A for major depressive disorder from the DSM. Exclusion criteria: major depressive episodes lasting more than two weeks.
**Undifferentiated risk group**	15–45 years old Subjects who complained of psychotic or mood symptoms not meeting the criteria above but meeting all of the following criteria: (1) symptoms experienced by the subject are causing distress and help-seeking, (2) cares where the clinician judges that the follow-up observation is necessary due to the possibility of future mental illness. Extended undifferentiated risk group. Meeting either of the following criteria: (1) Those who have been diagnosed with anxiety disorders during the last 5 years and (2) those with a family history of psychosis, bipolar disorders or anxiety disorders.

SOPS, Scale of Prodromal Symptoms; NOS, not otherwise specified; MDD, major depressive disorder.

### Procedure

**Figure 1:**
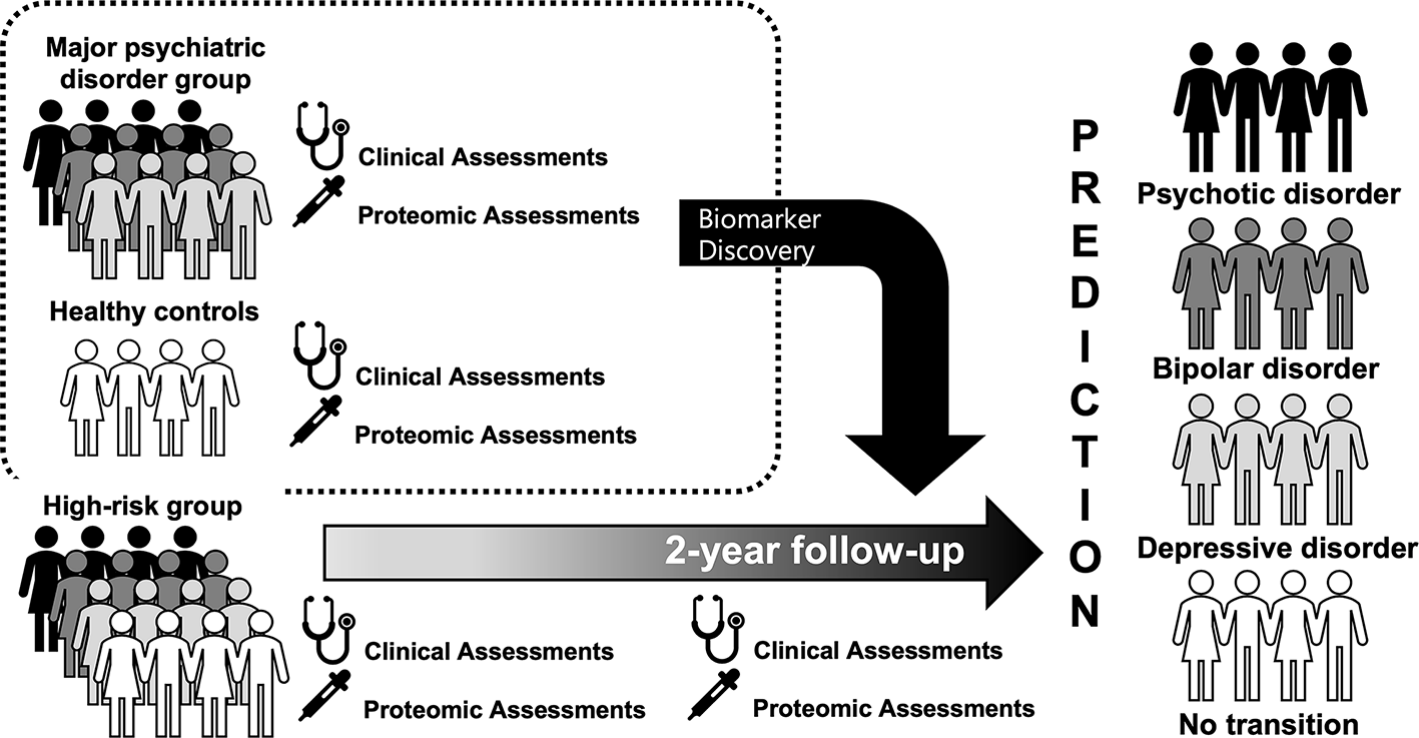
Flow chart showing the recruitment process for the patients and high-risk groups.

The first recruitment of high-risk subjects started in September 2017 and is expected to be completed in September 2019, and the follow-up evaluations will be continued until September 2021. After obtaining informed consent, a baseline interview is scheduled. If the subject is a minor, informed consent from their guardian is also obtained. A flow chart of the study procedure is shown in **Figure 1**. For the high-risk participants, they receive prospective follow-up evaluations, and the assessment points are at baseline, 6, 12, 18, and 24 months. A long-term follow-up investigation will continue at 6-month intervals if desired by the subjects, even if the 24-month follow-up period is over. All high-risk participants will receive case management and supportive psychotherapy on a bimonthly basis and will receive an intensive assessment within the 6-month intervals if they are expected to be converted or remitted. The conversion to a major mental illness and remission from the current high-risk status is determined by an intensive assessment and a consensus meeting. The definition of the transition was defined based on the diagnostic instruments 39, 40, and full remission was defined as no positive symptom item that met the severity corresponding to the criteria for a high-risk group and attribution criteria > 6 months. The patients with major mental disorders are also evaluated at baseline.

#### Clinical Assessments

At baseline, the high-risk participants are interviewed and diagnosed by a psychiatrist who has had many years of experience through the following diagnostic instruments: Structured Clinical Interview for the DSM-IV (SCID-I, II); Structured Interview for Prodromal Syndrome (SIPS) (39); and Bipolar Prodrome Symptom Scale-Prospective (BPSS-P) (40). To assess the clinical status and function at baseline, the following scales are performed: Positive and Negative Syndrome Scale (PANSS); Hamilton Depression Rating Scale (HAM-D) (41); Hamilton Anxiety Rating Scale (HAM-A) (42); Young's mania rating scale (YMRS) (43); Biological Rhythms Interview of Assessment in Neuropsychiatry (BRIAN) (44); Clinical Global Impression-Severity (CGI-S); Global Assessment of Functioning (GAF) (45); and Global Functioning: Social and Role Scales (GF) (46). The high-risk participants are also interviewed at the 6-, 12-, 18-, and 24-month follow-ups using the same instruments. For the patients with major mental disorders, they are also interviewed at baseline by the psychiatrist using the following diagnostic instruments: SCID-I, II; HAM-D; HAM-A; YMRS; CGI-S; GAF; and Brief Psychiatric Rating Scale (BPRS).

#### Self-Report Measures

At baseline, the high-risk participants complete the following self-report measures: Barratt Impulsiveness Scale (BIS) (47); Childhood Trauma Questionnaire (CTQ) (48); Morningness-Eveningness Questionnaire (MEQ) (49); WHO Quality of Life-BREF (WHOQOL-BREF) (50); Connor-Davidson Resilience Scale (CD-RISC) (51); Prodromal Questionnaire-Brief (PQ-B) (52); Quick Inventory of Depressive Symptomatology (QIDS) (53); Seasonal Pattern Assessment Questionnaire (SPAQ) (54); and State-Trait Anxiety Inventory (STAI) (55). The high-risk participants also complete the same self-report instruments at the 6-, 12-, 18-, and 24-month follow-up evaluations.

#### Proteomics Analysis

To elucidate potential blood biomarkers of (1) the disease-specific alterations in schizophrenia, bipolar disorder, and major depressive disorder and (2) the transition to major psychiatric disorders in high-risk groups, mass spectrometry-based proteomics analysis is performed in both the high-risk participants and the patients with major mental disorders. For protein identification and label-free quantification, mass spectra were processed, and peptide lists were searched against the human UniProt FASTA database (version 2014.10). After the plasma collection, sample preparations including protein digestion and peptide purification are performed as described above. Target proteins that are discovered in the major psychiatric disorder cohort are analyzed in the individual samples by liquid chromatography-tandem mass spectrometry (LC-MRM-MS) run using triple quadrupole (QQQ) mass spectrometry. These results are used as a template for each disease to validate the proteomic profiles in high-risk subjects. For proteomic analysis of blood biomarkers in a prospective cohort of transdiagnostic high-risk participants, targeted quantification is performed using multiple reaction monitoring (MRM) (56).

### Data Analyses

Descriptive statistics will be used to show the demographic and clinical characteristics of the participants at baseline and to examine the baseline characteristics in relation to the outcomes of the subjects. The Kaplan-Meier survival analysis will be used to examine the transition or remission in each high-risk group and the high-risk group as a whole. Cox regression will be used to determine the difference in hazard ratios between each high-risk group and in the high-risk group as a whole.

## Discussion

In the present study, we introduced the study protocol of the SPRIM study, which is the first prospective cohort study of transdiagnostic high-risk concepts using proteomic biomarkers. This study, an extension of studies of high-risk patients with psychosis that has lasted for 20 years, has a paradigm that encompasses various diseases without the aim of predicting and preventing the development of specific mental illnesses in help-seeking individuals. McGorry and his colleagues have already proposed a pluripotent prospective program that reflects this concept. The CHARM study encompasses schizophrenia, mood disorder, and borderline personality disorder outcomes and implements a clinical staging model along the mental health continuum (57). The CHARM study will also provide predictive and discriminant validity of the proposed criteria 16. Our study, on the other hand, focused on identifying the clinical characteristics in an Asian sample, and further look at predictors for blood biomarkers. Therefore, it is expected that the clinical usefulness of the transdiagnostic approach at an early stage will be tested. The transdiagnostic high-risk concept could be extended to provide a perspective for people with various psychopathological tendencies below a threshold, such that they do not meet the existing diagnostic criteria of mental illnesses, to determine what may lead them to a specific disease and help identify appropriate preventative interventions. Since the pluripotent high-risk group encompasses most mental illnesses, studies with large sample sizes will be needed to avoid false-negative consequences. In addition, further studies of other biomarkers that are receiving attention from psychosis will be needed (58–60). Further research into the pathophysiology leading to a risk for a specific mental illness in the pluripotent risk state and more common treatments such as cognitive behavioral therapy or anti-inflammatory therapy should be undertaken.

## Ethics Statement

The study was designed in accordance with the Declaration of Helsinki, and the protocol was approved by the Institutional Review Board of Seoul National University Hospital (no. 1704-075-846). Written informed consent will be obtained from the participants, or their parents when required.

## Author Contributions

All authors were responsible for the design of the whole study and wrote the protocol. Author TL wrote the manuscript. All authors supported the manuscript preparation and approved the final manuscript.

## Funding

This research was supported by the Brain Research Program through the National Research Foundation of Korea (NRF) funded by the Ministry of Science, ICT and Future Planning (Grant Nos. HI17C0870, 2019M3C7A1030625, 2019R1A2B5B03100844).

## Conflict of Interest

The authors declare that the research was conducted in the absence of any commercial or financial relationships that could be construed as a potential conflict of interest.
